# Alkali‐Ion‐Assisted Activation of ε‐VOPO_4_ as a Cathode Material for Mg‐Ion Batteries

**DOI:** 10.1002/advs.202307838

**Published:** 2024-05-06

**Authors:** Dogancan Sari, Ann Rutt, Jiyoon Kim, Qian Chen, Nathan T. Hahn, Haegyeom Kim, Kristin A. Persson, Gerbrand Ceder

**Affiliations:** ^1^ Department of Materials Science and Engineering Berkeley 94720 USA; ^2^ Materials Sciences Division Lawrence Berkeley National Laboratory Berkeley 94720 USA; ^3^ Material, Physical and Chemical Sciences Center Sandia National Laboratories Sandia 87185 USA

**Keywords:** cathodes, diffusion, energy storage, magnesium batteries, multivalent batteries

## Abstract

Rechargeable multivalent‐ion batteries are attractive alternatives to Li‐ion batteries to mitigate their issues with metal resources and metal anodes. However, many challenges remain before they can be practically used due to the low solid‐state mobility of multivalent ions. In this study, a promising material identified by high‐throughput computational screening is investigated, ε‐VOPO_4_, as a Mg cathode. The experimental and computational evaluation of ε‐VOPO_4_ suggests that it may provide an energy density of >200 Wh kg^−1^ based on the average voltage of a complete cycle, significantly more than that of well‐known Chevrel compounds. Furthermore, this study finds that Mg‐ion diffusion can be enhanced by co‐intercalation of Li or Na, pointing at interesting correlation dynamics of slow and fast ions.

## Graphical abstract

A novel cathode candidate for Mg‐ion batteries is revealed with the help of high‐throughput screening. The experimental and computational evaluation presented in this study provides a deeper understanding on promising electrochemical performance of ε‐VOPO4 and the dual cation effect.

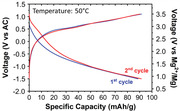

## 1 Introduction

In the last decades, Li‐ion batteries (LIBs) have undergone significant progress and contributed to revolutionary advances in many technologies, including energy‐dense portable devices and electric vehicles by providing both high energy and power.^[^ ^1^ ^]^ The remarkable growth of Li‐ion batteries is now hampered by resource concerns regarding high quality Li precursors and redox‐active transition metals including Co and Ni.^[^ ^2^, ^3^, ^4^ ^]^ Therefore, increasing research investment is being placed in the development of alternative battery systems. Multivalent (Mg, Ca, Al, and Zn) systems have been extensively studied for many years due to their potential for high volumetric capacity enabled by the metal anode, potential lower cost, and higher availability of essential components (Li‐, Co‐, and Ni‐free) compared to those of LIBs.^[^ ^5^ ^]^ Despite these numerous advantages, several challenges remain concerning the implementation of multivalent‐ion batteries. One such challenge is the poor diffusion of multivalent ions in solid phases at room temperature, necessitating the discovery of new material structures and chemistries that promote the mobility of multivalent ions.

Several research groups have introduced promising cathode candidates for Mg‐ion batteries.^[^ ^6^, ^7^, ^8^ ^]^ Some of these compounds, especially the sulfide‐ and selenide‐based cathodes, can provide specific capacities over 100 mAh g^−1^ (Mo_6_S_8_, Ti_2_S_4_, etc.); however, their low working voltage limits the energy content.^[^ ^9^, ^10^, ^11^, ^12^, ^13^, ^14^, ^15^ ^]^ Therefore, the exploration of new compounds that reversibly store Mg at a high voltage, and with a high capacity is necessary.

In this study, we identify ε‐VOPO_4_ as a material of interest using a previously reported high‐throughput computation screening method from the Materials Project Database,^[^ ^16^ ^]^ and experimentally evaluate its properties as a cathode material for Mg‐ion batteries. Moreover, the effects of the material's synthesis pathway and the presence of secondary mobile ions to assist Mg diffusion are investigated. We find that the ε‐VOPO_4_ cathode can provide a reversible specific capacity of 90 mAh g^−1^ with the average of the charge and discharge voltage ≈2.3 V versus Mg^2+^/Mg, which would result in a specific energy more than 200 Wh kg^−1^, higher than that of the well‐known Chevrel (Mo_6_S_8_) phase.

VOPO_4_ has seven distinct polymorphs: *α*_I_ (P4/n), *α*_II_ (P4/n), *β* (Pnma), δ (P42/mbc), ε (Cc), β (P42/mmc), and *ɣ* (Pbam).^[^ ^17^ ^]^ Among these, *α*_I_, *β*, and ε are easier to synthesize than the other polymorphs and, hence, have been the most thoroughly studied,^[^ ^17^ ^]^ with an emphasis on their electrochemical properties as multi‐electron redox cathodes in Li‐ion and Na‐ion batteries.^[^ ^18^ ^]^ The *α*_I_ polymorph is a layered structure composed of VO_5_–PO_4_ units. Several studies have investigated the *α*_I_ polymorph in which multivalent ion insertion may be enabled by increasing the interlayer spacing through the addition of water and/or phenylamine molecules.^[^ ^19^, ^20^, ^21^, ^22^ ^]^ The *β* and ε polymorphs are structurally similar, both consisting of a 3D network of VO_6_–PO_4_ units. Although *β*‐VOPO_4_ is more stable than ε‐VOPO_4_, both polymorphs can be experimentally accessed depending on the synthesis conditions.^[^ ^17^ ^]^ To the best of our knowledge, ε‐VOPO_4_ has never been studied as a cathode for Mg‐ion batteries.

## 2 Results

The ε‐VOPO_4_ (monoclinic, Cc) polymorph was identified from a computational screening ^[^ ^16^ ^]^ to find promising Mg cathode compounds. The computational evaluation used methods to predict voltage, capacity, ionic diffusivity, and stability of the end members, as widely used in the literature.^[^ ^16^, ^23^ ^]^ Only topotactic intercalation was considered as it exhibits better kinetics and cycling stability as compared to conversion‐type reactions (with a conversion voltage of 2.86 V) for ε‐VOPO_4_. The phase stability of ε‐VOPO_4_ (mp‐556459) was evaluated at a variety of Mg concentrations ^[^ ^24^ ^]^ using the convex hull method.^[^ ^25^ ^]^ The phase diagrams to construct the hull use stable structures in the Mg‐V‐O‐P space from the Materials Project ^[^ ^26^ ^]^ and the MP2020 Compatibility scheme.^[^ ^27^ ^]^ We found that the energy of ε‐VOPO_4_ is 3 meV per atom higher than that of the stable polymorph (*β*‐VOPO_4_) and that the Mg intercalated phase (ε‐Mg_0.5_VOPO_4_) is 25 meV per atom above the convex hull at 0 K. These relatively low energies above the ground state hull indicate that the Mg intercalated phase is likely to remain metastable and not decompose. The phase stability remains good when intercalation proceeds to ε‐MgVOPO_4_ which is only 14 meV per atom above the convex hull. Both Mg_0.5_VOPO_4_ and MgVOPO_4_ share the same symmetry with ε‐VOPO_4_ (space group Cc) with Mg ions occupying the *4a* Wyckoff sites, which become fully occupied in MgVOPO_4_. Further Mg insertion to Mg_1.5_VOPO_4_ in additional crystallographic sites is feasible in principle but makes the compound very unstable putting it at 164 meV per atom above the convex hull. In addition, when x > 1 in ε‐Mg_x_VOPO_4_, the valence state of V will drop below +3, which will likely occur at very low voltage, making this region less attractive. Therefore, only calculations for ε‐Mg_x<1_VOPO_4_ are further included in this study.

**Figure 1:**
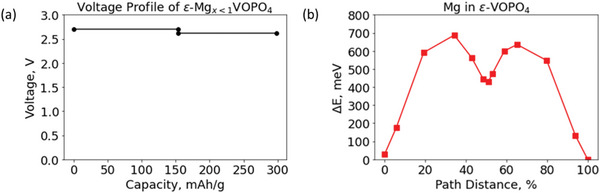
a) Capacity versus voltage plot of ε‐VOPO_4_ ↔ ε‐Mg_0.5_VOPO_4_ and ε‐Mg_0.5_VOPO_4_ ↔ ε‐MgVOPO_4_ predicted by DFT, b) Climbing image ‐nudged elastic band (CI‐NEB) calculations showing migration barrier for Mg^2+^ ions in ε‐VOPO_4_.

**Figure** **1**a presents the computed voltage profile of ε‐VOPO_4_ up to the intercalation of one Mg^2+^ per formula unit going through the V^5+/4+/3+^ redox reaction. The computation predicts a voltage of 2.7 V (vs Mg/Mg^2+^) for ε‐V^5+^OPO_4_ ↔ ε‐Mg_0.5_V^4+^OPO_4_ with a capacity of 165 mAh g^−1^, followed by a voltage of 2.6 V (vs Mg/Mg^2+^) for ε‐Mg_0.5_V^4+^OPO_4_ ↔ ε‐MgV^3+^OPO_4_ with an additional capacity of 165 mAh g^−1^. We note that the voltages for the two stages are close (2.7 V vs 2.6 V), which is in contrast of what has been found for monovalent ion intercalation in ε‐VOPO_4_ and VPO_4_F, for example.^[^ ^17^, ^28^ ^]^ We believe that the large voltage drop in these systems is mainly due to the high instability of A_2_VOPO_4_ when the second Li/Na is inserted. In divalent systems, the V^4+^ state is reached with only half the number of working ions intercalated and we find that Mg_0.5_VOPO_4_ and MgVOPO_4_ exhibit similar stability, leading to similar voltages between VOPO_4_ to Mg_0.5_VOPO_4_ and Mg_0.5_VOPO_4_ to MgVOPO_4_.

To evaluate the Mg mobility, we computed the Mg^2+^ migration barrier in the dilute limit (a single Mg ion in 2 × 2 × 2 supercells equivalent to one Mg per 16 VOPO_4_ formula units) of the ε‐VOPO_4_ host structure. As shown in Figure 1b, the CI‐NEB calculation indicates a Mg^2+^ migration barrier of 687 meV. We estimate that the 687‐meV barrier for Mg^2+^ migration would result in a diffusivity of ≈4 × 10^−15^ cm^2^ s^−1^ using the method proposed by Rong et al.^[^ ^29^ ^]^ This diffusivity would be sufficient for at least a C/8 cycling rate at room temperature with 100‐nm‐sized particles according to the report by Rong et al.^[^ ^29^ ^]^ The migration barrier is larger than NEB barriers found for divalent ions in the layered VOPO_4_, for example, 0.4 eV for Mg in hydrated VOPO_4_^[^ ^19^ ^]^ and 0.45 eV for Ca.^[^ ^30^ ^]^ We also performed NEB calculations with Li or Na in ε‐VOPO_4_ in the dilute limit along the same hopping pathway (Figure S6, Supporting Information). Both Li and Na show a lower migration barrier (288 and 456 meV) than Mg, suggesting they diffuse faster in the same framework. The fast diffusion of Li and Na in ε‐VOPO_4_ may promote Mg diffusion and lead to the better capacity in the Li/Na pre‐cycled sample as has been found in several systems where fast ions were found to improve the diffusion of the slower ion.^[^ ^31^, ^32^ ^]^ To assess the Mg mobility in ε‐VOPO_4_ at higher Mg concentration, we performed NEB calculation for the same path in the limit where one vacancy is present in the 2 × 2 × 2 ε‐MgVOPO_4_ supercells. The results are shown in Figure S7 (Supporting Information). The vacancy hop shows a barrier of 366 meV, lower than that in the dilute Mg limit.

**Figure 2:**
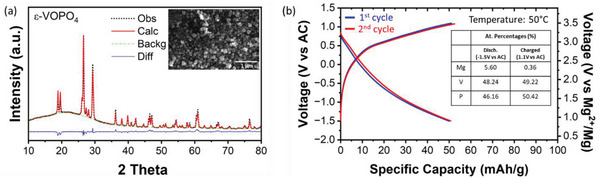
a) XRD pattern (inset: SEM image) of ε‐VOPO_4_ synthesized via a hydrothermal method. b) Electrochemical cycling data of hydrothermally synthesized ε‐VOPO_4_ in a cell with activated carbon anode and 0.5 m Mg(TFSI)_2_ / 1 m diglyme / TTE electrolyte. (Inset: composition of ex situ samples collected after a full discharge and full charge). (Refinement parameters for part (a); Rexp = 0.65, Rwp = 1.1, χ 2 = 2.86).

To experimentally evaluate ε‐VOPO_4_ as a Mg intercalation cathode, we synthesized ε‐VOPO_4_ via a hydrothermal method, following procedures in the literature ^[^ ^18^ ^]^ (see the Experimental Section for details). **Figure** **2**a presents the X‐Ray diffraction (XRD) pattern of the synthesized ε‐VOPO_4_ (monoclinic, Cc) and the refinement results. The refined lattice parameters are *a* = 7.266 Å, *b* = 6.891 Å, *c* = 7.263 Å, and *β* = 117°, in good agreement with the literature.^[^ ^18^, ^33^ ^]^ We did not observe any impurity phases in the XRD pattern. The inset of Figure 2a presents an scanning electron microscopy (SEM) image of ε‐VOPO_4_ in which ≈100‐nm particles are homogeneously distributed.

Figure 2b presents galvanostatic charge–discharge profiles of the ε‐VOPO_4_ cathode in a two‐electrode coin‐cell configuration at 50 °C. In this electrochemical test, activated carbon (AC) was used as a counter electrode because of its superior stability against the electrolyte compared with Mg metal. Because the AC counter electrode stores charge via capacitive reactions with a linear voltage increase upon charging,^[^ ^7^ ^]^ we used excess loading of the AC counter electrode with more than 20 times excess mass compared with that of the working electrode of ε‐VOPO_4_. Because of the high working electrode/counter electrode ratio, the counter electrode should not limit the capacity of ε‐VOPO_4_ upon Mg^2+^ intercalation while also allowing us to minimize the effect of the counter electrode in the estimation of the potential versus Mg/Mg^2+^ by producing an almost constant voltage of the AC.

The ε‐VOPO_4_ cathode delivered a reversible specific capacity of 50 mAh g^−1^ at a current density of 2 mA g^−1^ and an average voltage of 1.7 V versus Mg/Mg^2+^ upon discharging and 2.9 V versus Mg/Mg^2+^ upon charging. The discrepancy between the experimental‐measured and theoretical voltages can be attributed to the polarization caused by the limited Mg ion diffusivity in ε‐VOPO_4_ as well as the fact that the voltage was converted to the voltage versus Mg/Mg2+ from versus AC. To confirm Mg^2+^ intercalation into the ε‐VOPO_4_ cathode, we conducted ex situ energy dispersive spectroscopy (EDS) analysis at fully discharged and fully charged conditions, as shown in the inset of Figure 2b. The discharged and charged samples show Mg/V atomic ratios of 0.12 and 0.007, respectively. Although a slightly lower Mg content (Mg_0.12_VOPO_4_) was determined from EDS quantification than that estimated from the discharge capacity (Mg_0.15_VOPO_4_), the result demonstrates that the Mg^2+^ (de)intercalation reaction is responsible for the capacity of the ε‐VOPO_4_ cathode. It is possible that some of the Mg^2+^ in the ε‐VOPO_4_ structure was washed away during the ex situ sample preparation.

Recently, it has been reported that the presence of secondary mobile ions in the host structure can improve the cycling kinetics and stability.^[^ ^31^, ^32^, ^34^ ^]^ Inspired by these studies, we investigated the effect of precycling the cathode with Li^+^ on the Mg^2+^ intercalation into ε‐VOPO_4_. We selected ε‐LiVOPO_4_ as a model system because the ε‐VOPO_4_ structure is known to form after the removal of Li. The ε‐LiVOPO_4_ phase was synthesized by a sol‐gel method (see Experimental Section). We confirmed that the ε‐LiVOPO_4_ phase was successfully synthesized without noticeable impurities such as *β*‐LiVOPO_4_ or Li_3_V_2_(PO_4_)_3_, which often form as a secondary phase in the synthesis (Figure S1a, Supporting Information).

The ε‐LiVOPO_4_ cathode was placed into a Li cell for the delithiation process. A charge capacity of 151 mAh g^−1^ was achieved (Figure S2, Supporting Information), which is ≈95% of the theoretical capacity (158.7 mAh g^−1^ for complete delithiation). After completing the charging, the cell was disassembled, and the cathode was removed and washed in ethylene carbonate/diethyl carbonate (EC/DEC) solvent to remove any residual salt on the surface of the cathode film. All these procedures were conducted in an Ar‐filled glovebox. Although the charge capacity in the Li cell is very close to the theoretical capacity of ε‐LiVOPO_4_, we expect that a small amount of Li ions remains in the structure. Hence, we refer to the material as ε–Li_x_VOPO_4_ in the following sections. We confirmed that the XRD patterns collected after the electrochemical delithiation process matched well with the ε‐VOPO_4_ phase without any secondary phases (Figure S1c, Supporting Information). Although the particle size of ε‐LiVOPO_4_ is not uniform and has a bimodal distribution (≈100 nm and ≈1 µm), the large particles are reduced to 100–200 nm after delithiation, which might originate from the volume change during the Li extraction (Figure S1b,d, Supporting Information).

**Figure 3:**
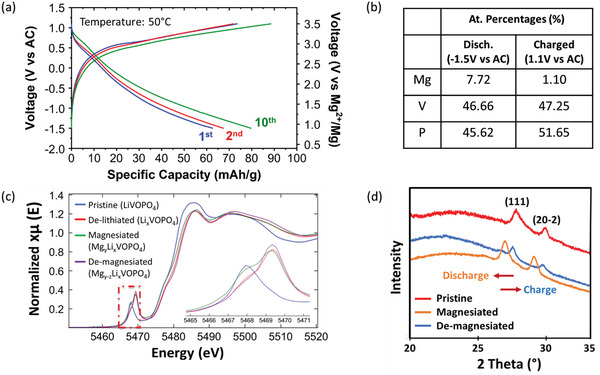
(a) Electrochemical cycling data (1st, 2nd, and 10th charge and discharge curves) of ε‐Li_x_VOPO_4_ in a cell with AC anode and 0.5 m Mg(TFSI)_2_ / 1 m diglyme / TTE electrolyte. b) Quantitative EDS results after discharge and charge in a Mg cell. (c) Vanadium K‐edge X‐Ray absorption spectra (XAS) of pristine, de‐lithiated, magnesiated, and de‐magnesiated ε‐Li_x_VOPO_4_. (The inset shows an enlarged image of the pre‐edge region.) d) XRD patterns of ε‐Li_x_VOPO_4_ (collected from the cathode films before Mg cycling (red), after Mg insertion (orange), and after Mg removal (blue)) showing reversible peak shifts during Mg insertion (lattice expansion) and removal (lattice contraction).

**Figure** **3**a presents galvanostatic charge–discharge profiles of ε‐Li_x_VOPO_4_ in Mg cells after delithiation. In our experiments, the ε‐Li_x_VOPO_4_ cathode was cycled against an AC counter electrode and 0.5 m Mg(TFSI)_2_ / 1 m diglyme / TTE electrolyte at 50 °C with a current density of 2 mA g^−1^. The ε‐Li_x_VOPO_4_ cathode shows a first discharge capacity of 63 mAh g^−1^, which corresponds to Mg_0.16_VOPO_4_. This is slightly higher than the first discharge capacity of ε‐VOPO_4_ without precycling in a Li cell (50 mAh g^−1^) in Figure 2. In successive cycles, the discharge capacity gradually increases up to 80 mAh g^−1^, corresponding to Mg_0.24_VOPO_4_. We further confirmed the amounts of Mg/V after the first discharge and charge processes using EDS, as shown in Figure 3b. The Mg content at the end of the first discharge (Mg/V = ≈0.16) is higher than that of the directly synthesized ε‐VOPO_4_ (Mg/V = ≈0.12) in Figure 2.

We employed ex situ XAS and XRD experiments to understand the structure change and redox mechanism in ε‐Li*_x_* VOPO_4_ upon charging and discharging in Mg cells. Figure 3c presents the V K‐edge X‐Ray absorption near‐edge structure (XANES) analysis results of ε‐Li*_x_* VOPO_4_ after discharge and charge in Mg cells along with pristine ε‐LiVOPO_4_ and delithiated ε‐Li*_x_* VOPO_4_. The V pre‐edge peak in the as‐synthesized (pristine) ε‐LiVOPO_4_ is observed at ≈5468 eV, indicating the presence of V^4+^ in the compound. After the electrochemical charging in a Li cell, vanadium ions are oxidized to V^5+^, as evidenced by the shift of the pre‐edge peak to higher energy, 5469.5 eV. We also confirmed that upon Mg^2+^ intercalation the V pre‐edge peak shifts to lower energy, 5468 eV, indicating a partial reduction of V^5+^ to V^4+^. Charging the sample causes the V pre‐edge peak to return to the high‐energy position at ≈5469.5 eV, indicating the oxidation of V^4+^ to V^5+^. These results confirm that the Mg^2+^ intercalation and deintercalation processes are accompanied by the reversible reduction and oxidation of the vanadium ions. Figure 3d presents the XRD patterns of ε‐Li*_x_* VOPO_4_ after discharge and charge in Mg cells. The XRD peaks shift to lower angles after discharge and return to their original positions in the charged state, indicating topotactic Mg^2+^ intercalation and deintercalation. It should be noted that the XRD peaks have lower signal to noise ratio and more peak broadening in the ex situ samples (Figure 3d) compared to the as‐synthesized XRD pattern (Figure 2a) due to lower amount of active material in the ex situ samples (≈2 mg cm^−2^ per cathode film) compared to the as‐synthesized powder (≈30 mg cm^−2^).

**Figure 4:**
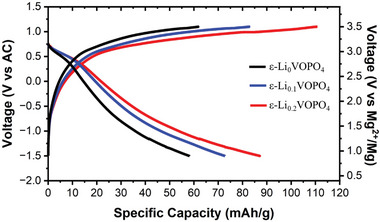
Electrochemical cycling data of hydrothermally synthesized ε‐VOPO_4_ with the approximate compositions of ε‐Li_0_VOPO_4_ (black), ε‐Li_0.1_VOPO_4_ (blue), and ε‐Li_0.2_VOPO_4_ (red) in a cell with AC anode and 0.5 m Mg(TFSI)_2_ / 1 m diglyme / TTE electrolyte.

To better understand the effect of remaining Li ions in the ε‐VOPO_4_ structure, we intentionally intercalated varied Li‐ion contents in hydrothermally synthesized ε‐VOPO_4_ in a Li half‐cell. By this technique, we targeted to exclude the effects other than the Li content (such as average particle size and size distribution) in the electrochemical performance of the ε‐VOPO_4_. In these experiments, all samples were cycled at room temperature at a current density of 5 mA g^−1^ for three cycles. One of the cathodes was recovered after the 3rd cycle in a fully charged condition, ε‐Li_0_VOPO_4_. The other two cells were partially discharged at the end of the 3rd cycle. The discharge processes of these cells were stopped at different specific capacities to obtain the following compositions: ε‐Li_0.1_VOPO_4_ and ε‐Li_0.2_VOPO_4_. All three cathode samples were recovered from the coin cells, washed in EC/DEC solvent to remove remaining salts, and dried in an Ar‐filled glovebox. **Figure** **4** presents the charge–discharge profiles of the three cathodes, ε‐Li_0_VOPO_4_, Li_0.1_VOPO_4_, and ε‐Li_0.2_VOPO_4_ in Mg cells. Interestingly, the specific capacities increased linearly in response to increasing Li content in the compounds. The highest discharge capacity of 87 mAh g^−1^ was obtained for Li_0.2_VOPO_4_, whereas the first discharge capacity of ε‐Li_0_VOPO_4_ was measured to be only 59 mAh g^−1^. The same trend was observed in the charge capacities of the samples: 62, 84, and 111 mAh g^−1^ for ε‐Li_0_VOPO_4_, ε‐Li_0.1_VOPO_4_, and ε‐Li_0.2_VOPO_4_, respectively. Since the irreversible charge capacities indicate the increased amount of side reactions, we did not increase the Li content above ε‐Li_0.2_VOPO_4_. These results clearly demonstrate that the pre‐intercalated Li ions in ε‐VOPO_4_ are beneficial for Mg^2+^ intercalation.

We further investigated whether pre‐cycling with Na^+^ similarly causes an improvement in Mg^2+^ intercalation into ε‐VOPO_4_ because we expect that the pre‐intercalation of the larger Na ions (relative to the Li ions) might expand the lattice volume slightly and help the Mg^2+^ intercalation kinetics. In this experiment, the ε‐VOPO_4_ cathode was cycled in a Na cell at 2 mA g^−1^ at room temperature (Figure S3, Supporting Information) prior to electrochemical cycling in a Mg cell.

**Figure 5:**
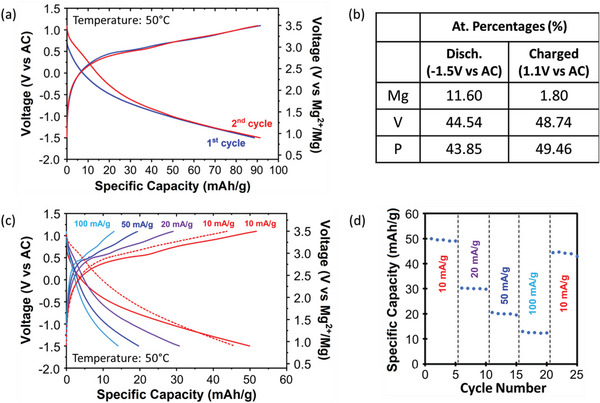
a) Electrochemical cycling data of Na activated ε‐VOPO_4_ in a cell with AC anode and 0.5 m Mg(TFSI)_2_ / 1 m diglyme / TTE electrolyte. b) Quantitative EDS results after discharge and charge in a Mg cell. c) Discharge and charge profiles of the first cycles with different rates. d) Capacity retention with cycling at varied current rates.

**Figure** **5**a presents the charge–discharge profiles of the ε‐VOPO_4_ cathode after Na pre‐cycling. The first discharge capacity is 89 mAh g^−1^, which is significantly higher than that of the ε‐VOPO_4_ sample that was tested directly in Mg cells (Figure 2b) and even higher than that of the ε‐VOPO_4_ sample that was pre‐cycled in a Li cell (Figure 3a). The compositions of the samples after complete discharge (Mg_0.26_VOPO_4_) and complete charge (Mg_0.04_VOPO_4_) were measured using EDS (Figure 5b). These compositional changes in Mg content are in good agreement with the observed discharge and charge capacity. It should also be noted that the voltage curves during discharge and charge appeared closer to each other in the Na pre‐cycled samples than in the samples that were tested directly in Mg cells, indicating improved Mg^2+^ intercalation kinetics.

Figure 5c,d shows the rate capability of the Na‐precycled ε‐VOPO_4_ cathode. We cycled the Na‐precycled ε‐VOPO_4_ cathode at varied current rates from 10 to 100 mA g^−1^. At each current rate, the Na‐precycled ε‐VOPO_4_ cathode was tested for five cycles. The first discharge capacity of 51 mAh g^−1^ was measured at a rate of 10 mA g^−1^. The specific capacity decreases to 28, 19, and 13 mAh g^−1^ when the current rate increases to 20, 50, and 100 mA g^−1^, respectively (Figure 5c). After the rate capability test up to 100 mA g^−1^, the current rate was reduced back to 10 mA g^−1^ (dashed line in Figure 5c), with a recovered capacity of 45 mAh g^−1^, indicating good cycling stability. The stability of the cathode can also be seen in Figure 5d, where 25 complete cycles were applied, and the compound retained more than 90% of its initial capacity.

## 3 Discussion

**Figure 6:**
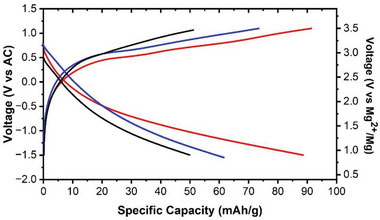
Electrochemical cycling data of ε‐VOPO_4_ without pre‐cycling (black), Li‐precycled ε‐VOPO_4_ (blue), and Na‐precycled ε‐VOPO_4_ (red) in a cell with AC anode and 0.5 m Mg(TFSI)_2_ / 1 m diglyme / TTE electrolyte.

In this work, ε‐VOPO_4_, identified by a high‐throughput computational screening method from the Materials Project Database, is investigated as a promising Mg‐cathode. CI‐NEB calculations revealed a migration barrier of 687 meV. In the literature, migration barriers that are below 800 meV are mainly observed in layered structures including *α*_1_‐VOPO_4_. As ε‐VOPO_4_ does not have a layered structure, it offers a new perspective in the search for new Mg‐cathodes. The preliminary experimental evaluation of ε‐VOPO_4_ resulted in a reasonable reversible capacity (50 mAh g^−1^) at 50 °C. The subsequent experiments proved the beneficial effect of the precycling with Li and Na on the performance of Mg intercalation and deintercalation of ε‐VOPO_4_. **Figure** **6** presents the voltage profiles of the initial cycles of ε‐VOPO_4_ without pre‐cycling (dash‐dot, black), Li‐precycled ε‐VOPO_4_ (dash, blue), and Na‐precycled ε‐VOPO_4_ (solid, red), with specific capacities of 50, 62, and 89 mAh g^−1^ obtained, respectively. In addition to the increased capacities, the voltage profiles show less overpotential for the discharge and charge processes in the Li‐precycled and Na‐precycled samples.

The dual cation effect has been observed first in layered cathodes where the presence of the slower ion helps to stabilize the structure and allows faster diffusion kinetics for the faster ion.^[^ ^31^ ^]^ The results of the current study show the remarkable effect that the presence of fast ions (such as Li^+^ and Na^+^) may assist the diffusion of the slower Mg^2+^ ion as well, similar to what has been observed in Na‐containing NASICONs where the presence of Na assists the diffusion of Ca.^[^ ^32^ ^]^ Such concerted motion of distinct ions is an exciting new opportunity to enhance mobility of divalent ions.

## 4 Conclusion

In summary, a high‐throughput screening was conducted, and promising energy‐density and migration barriers were revealed for the ε‐VOPO_4_ compound with the help of DFT calculations. We predicted an average voltage of 2.7 V and a capacity of 288 mAh g^−1^ for ε‐VOPO_4_ ↔ ε‐MgVOPO_4_. NEB simulations predicted a migration barrier of 694 meV for Mg^2+^ ions in ε‐VOPO_4_. We further experimentally proved that the ε‐VOPO_4_ can work as a Mg cathode with reversible capacities reaching up to 90 mAh g^−1^ with an average voltage of −0.1 V versus AC (≈2.3 V vs Mg^2+^/Mg). The study also supports the beneficial effect of Li and/or Na precycling for the electrochemical performance of ε‐VOPO_4_ cathodes in Mg cells.

## 5 Experimental Section

### Computational Methods

All DFT calculations were performed with the Vienna Ab initio Software Package (VASP). The exchange correlation was approximated with the Perdew–Burke–Ernzerhof (PBE) generalized gradient approximation (GGA). A Hubbard U correction on V of U = 3.25 eV was used (consistent with “MPRelaxSet” in pymatgen ^[^ ^35^ ^]^), as GGA was known for underestimating the voltage due to the incomplete cancellation of the self‐interaction in the *d*‐orbitals.^[^ ^35^, ^36^ ^]^ Pseudopotentials were also selected according to “MPRelaxSet” specified in pymatgen.^[^ ^35^ ^]^ The total energy was sampled using a Monkhorst–Pack mesh with a *k*‐point density of 64 Å^−3^. Projector augmented‐wave theory combined with a well‐converged plane‐wave cutoff of 520 eV were used to describe the wave functions. The convergence threshold of the total energy was set to 0.00005 eV per atom and a force tolerance of 0.05 eV Å^−1^.

Nudged elastic band (NEB) calculations were performed in 2 × 2 × 2 supercells at the dilute lattice limit to better characterize the ion mobility. These cells contain 113 atoms (1 Mg per 16 VOPO_4_ formula units) and have all lattice parameters > 10 Å to avoid fictious self‐interaction effects due to periodic boundary conditions. Calculation parameters consistent with “MPRelaxSet” specified in pymatgen were adopted except for the following changes. A Hubbard U correction was not included in these calculations as there was no conclusive evidence that GGA+U performs better when investigating ion migration with NEB.^[^ ^37^, ^38^, ^39^, ^40^ ^]^ Gaussian smearing was used. No symmetry but Ψ_k_ = Ψ^*^_‐k_ was assumed to reduce sampling of the Brillouin zone. An additional support grid for the evaluation of the augmentation charge was applied. A minimum of four electronic self‐consistency steps was required. The end‐point structures converged with 0.00005 eV and 0.01 eV Å^−1^ cut‐off criteria during their relaxations. A linear interpolation of five images was used between relaxed end points. The images were converged to 0.00005 eV and 0.05 eV Å^−1^ cut‐off criteria for the NEB calculation. After the first NEB calculation, the intermediate local energy minimum image was relaxed as an end point to break up the pathway into two segments for a more refined climbing image NEB (CI‐NEB) calculation. In CI‐NEB, the images were converged to 0.00005 eV and 0.01 eV Å^−1^.

### Synthesis

Synthesis of ε‐VOPO_4_ was performed using a hydrothermal synthesis method.^[^ ^18^ ^]^ Stoichiometric ratios of VCl_3_ and P_2_O_5_ (Sigma–Aldrich) were used as the precursors, which were dissolved in ethanol. The solution was heated to 180 °C for 3 days in an autoclave to obtain monoclinic H_2_VOPO_4_. The resulting powder was collected by centrifuge and dried for 12 h. Then, the powder was grinded, pressed, and heated to 550 °C for 3 h under constant oxygen flow, which resulted in high‐purity ε‐VOPO_4_.

The sol‐gel technique was used to obtain *α*‐LiVOPO_4_,^[^ ^41^ ^]^ which forms ε‐VOPO_4_ upon electrochemical charging in a Li cell. To attain *α*‐LiVOPO_4_, 405 mg of NH_4_VO_3_, 395 mg of NH_4_H_2_PO_4_, 89 mg of LiF, and 0.864 mL of hexanoic acid (CH_3_(CH_2_)_4_COOH) were mixed in 150 mL of H_2_O for 24 h and then heated to 80 °C to evaporate the solvent. The remaining yellow mixture was pressed and calcined at 300 °C for 3 h under Ar flow. After calcination, the product was grinded and pressed again into pellet form and sintered at 650 °C for 4 h under Ar flow.

### Electrochemistry

The active material, carbon black (Timcal, SUPER C65), and polytetrafluoroethylene (PTFE; DuPont, Teflon 8A) were mixed in a glovebox at a weight ratio of 7:2:1 to prepare the cathode films. The anodes were prepared by mixing AC (Sigma), carbon black, and PTFE at a weight ratio of 8:1:1 in the glovebox. The mixtures were rolled to form thin‐film cathodes and anodes. The coin cells were prepared by using these cathode and anode thin films with a loading density of 3 mg cm^−2^ cathode to 20 mg cm^−2^ anode.

The electrolyte was prepared by drying magnesium(II) bis(trifluoromethanesulfonyl)imide (Mg(TFSI)_2_; 99.5%, Solvionic) salt at 170 °C overnight in an Ar‐filled glovebox. Then, the dried salt was used to form 0.5 m Mg(TFSI)_2_ + 1 m diglyme (99.5%, Sigma–Aldrich) solution in 1,1,2,2‐tetrafluoroethyl‐2,2,3,3‐tetrafluoropropyl ether (TTE; TCI Chemicals). The water content of the electrolyte was measured by Karl‐Fischer titration technique and found to be lower than 10 ppm. This electrolyte was selected to obtain higher oxidative stability than the standard glyme‐based electrolytes.^[^ ^42^ ^]^ The electrolyte and its components were always kept in the glovebox.

Coin cells were assembled using the electrolyte, cathode, and anode thin films described above and separators (Whatman glass microfiber filter). Galvanostatic cycling tests were performed at 50 °C using an Arbin battery tester. The tests were conducted at a current density of 2 mA g^−1^, and ex situ samples were collected after washing the cathode thin films with diglyme in an Ar‐filled glovebox.

### Characterization

The phase identification of the synthesized samples and the structural changes in the cathodes were observed by ex situ XRD using a Rigaku MiniFlex 600 diffractometer with Cu Kα radiation (*λ* = 1.54178 Å) in the 2*θ* range of 10°–80°. Rietveld refinement was performed using the PANalytical X'Pert HighScore Plus software. EDS analysis was performed, and SEM images were collected using a Zeiss Gemini Ultra‐55 analytical field‐emission SEM at the Molecular Foundry at Lawrence Berkeley National Lab (LBNL).

V K‐edge XAS measurements were performed at the Advanced Photon Source, Argonne National Laboratory. All the ex situ samples were sealed between polyimide (Kapton) tape to prevent air exposure. The XAS spectra were calibrated and normalized using the Athena software package.

## Conflict of Interest

The authors declare no conflict of interest.

## Supporting information

Supporting Information

## Data Availability

The data that support the findings of this study are available from the corresponding author upon reasonable request.
