# Supplementary material for: Alkali‐Ion‐Assisted Activation of ε‐VOPO4 as a Cathode Material for Mg‐Ion Batteries
Source: Adv Sci (Weinh). 2024 May 6;11(26):2307838. doi: 10.1002/advs.202307838 (PMC11234458; doi:10.1002/advs.202307838)
Supplement: Supplementary file 1 — Supporting Information [file ADVS-11-2307838-s001.pdf]

ADVANCED SCIENCE
Open Access

Supporting Information

for *Adv. Sci.*, DOI 10.1002/advs.202307838

Alkali-Ion-Assisted Activation of ϵ-$VOPO_4$ as a Cathode Material for Mg-Ion Batteries

Dogancan Sari, Ann Rutt, Jiyoon Kim, Qian Chen, Nathan T. Hahn, Haegyeom Kim, Kristin A. Persson* and Gerbrand Ceder**

Supporting Information

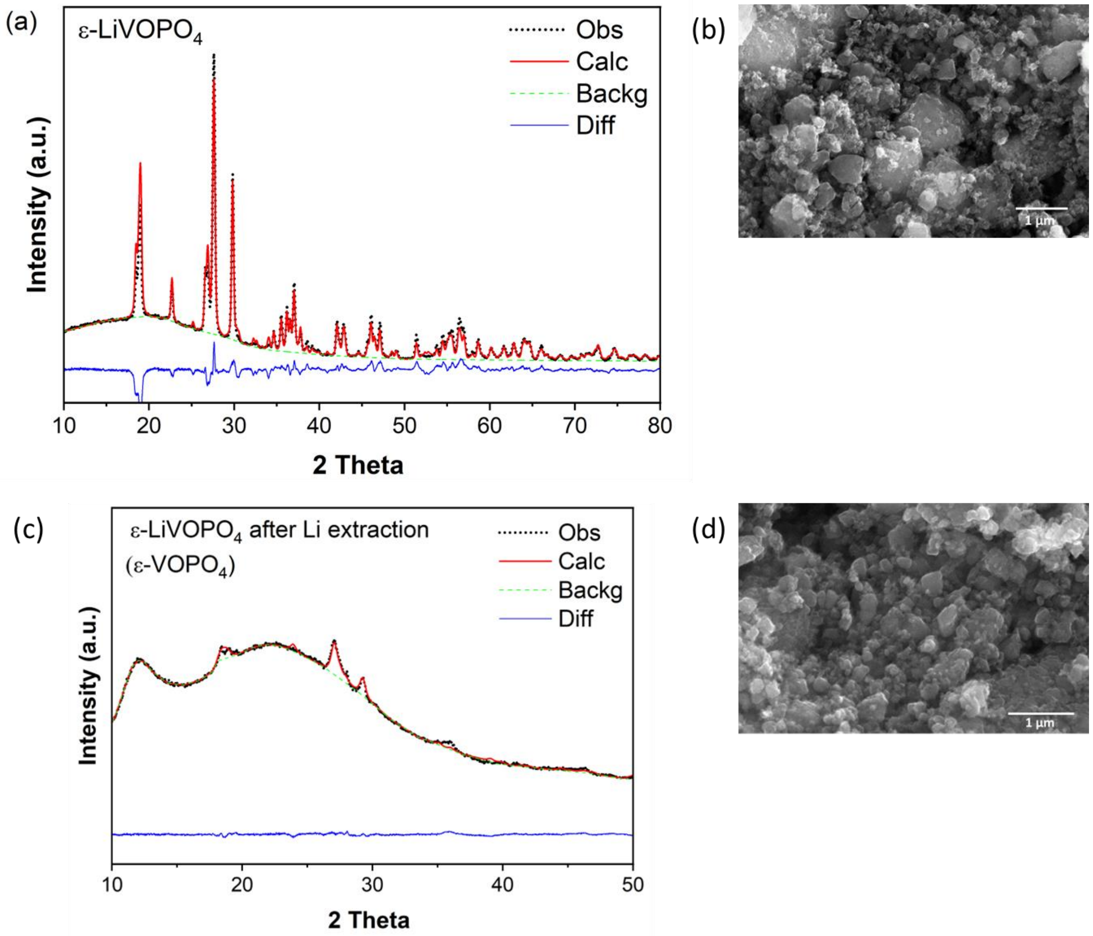

Figure S1. XRD patterns and SEM images of ε-$LiVOPO_4$ synthesized via sol-gel method (a, b) before and (c, d) after Li extraction. (Rietveld refinement parameters for part (a) Rexp = 0.68, Rwp = 2.87, χ 2 = 4.24)

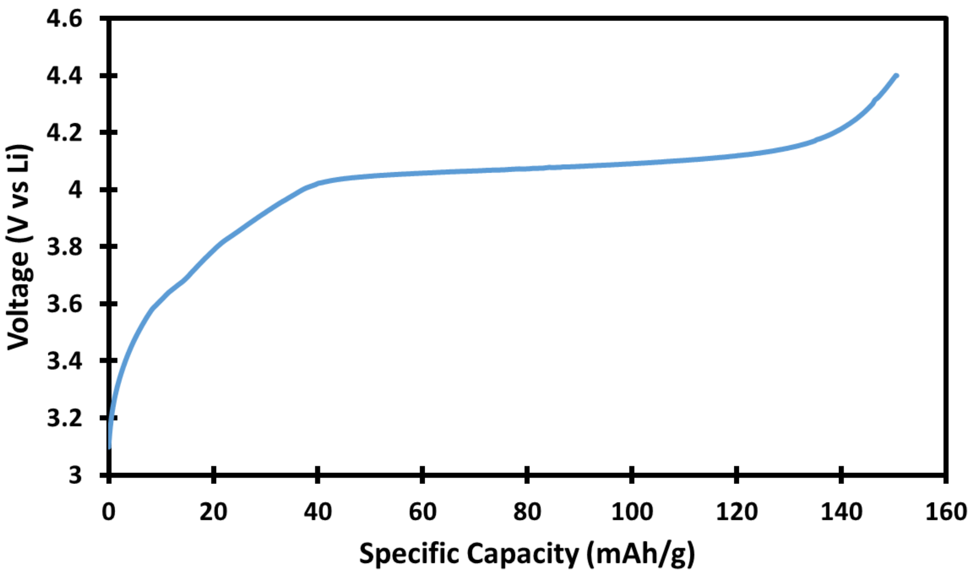

Figure S2. Charging voltage–capacity profile of α-Li$VOPO_4$ sample in a cell with Li foil anode and 1 M LiPF6/EC:DEC electrolyte.

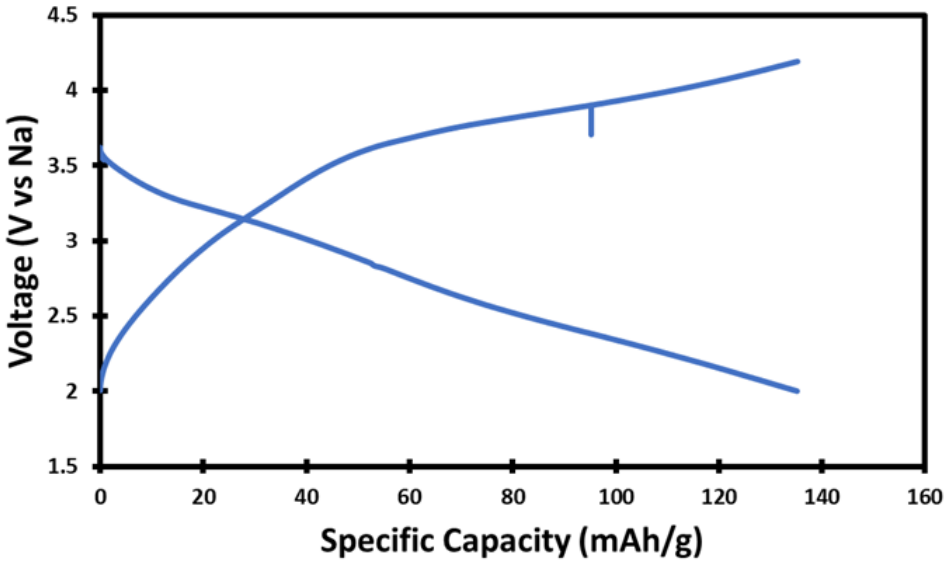

Figure S3. Electrochemical cycling data of hydrothermally synthesized ε-$VOPO_4$ in a cell with Na metal foil as the anode and 1 M NaPF6/EC:DEC electrolyte.

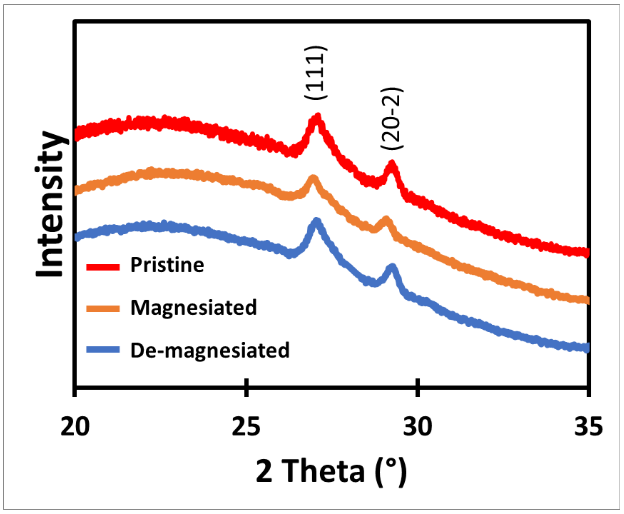

Figure S4. XRD patterns of ε-$VOPO_4$ (collected from the cathode films before Mg cycling (red), after Mg insertion (orange) and after Mg removal (blue)) showing reversible peak shifts during Mg insertion (lattice expansion) and removal (lattice contraction).

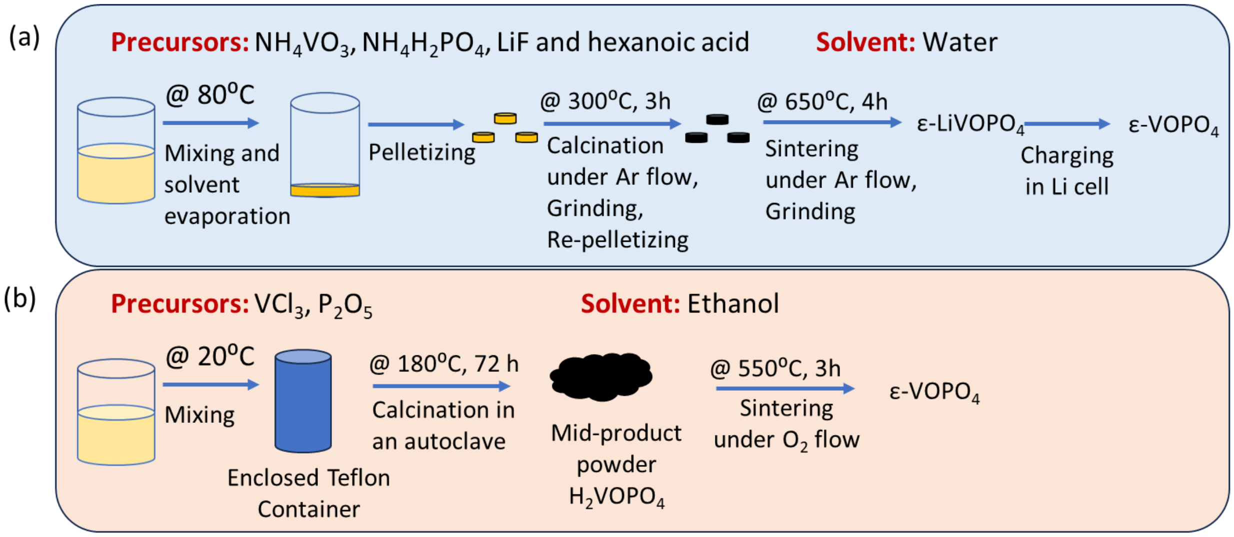

Figure S5. Flow charts of the synthesis procedure of ε-$VOPO_4$ via (a) sol-gel synthesis and (b) hydrothermal synthesis technique.

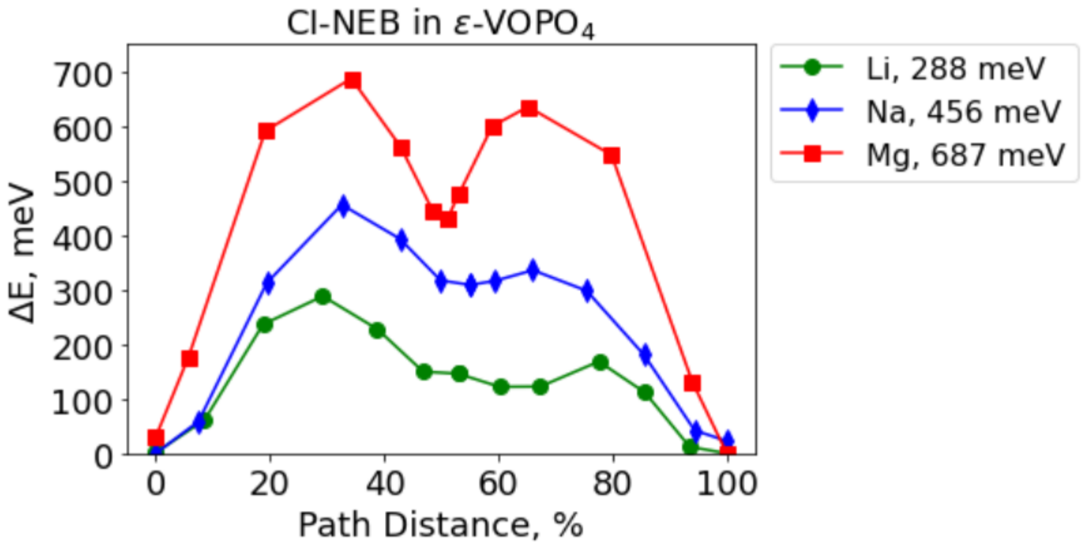

Figure S6. NEB calculations showing migration barrier for Li/Na/Mg in ε-$VOPO_4$.

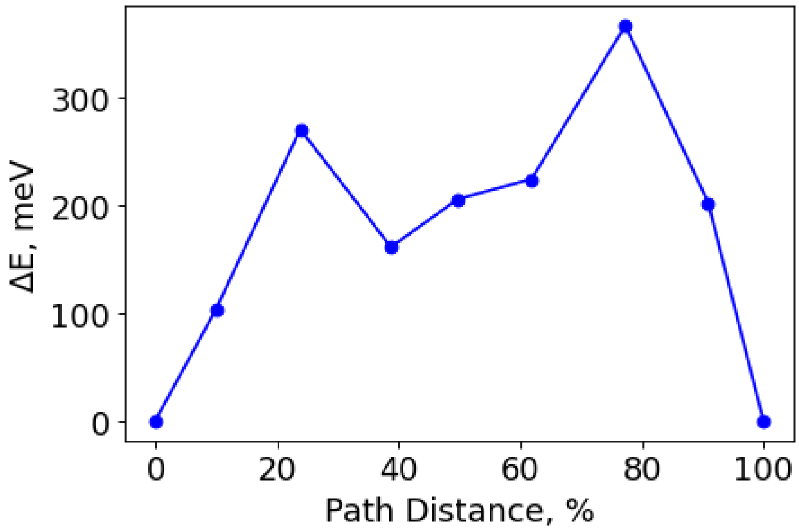

Figure S7. NEB calculations showing migration barrier for a vacancy in ε-$MgVOPO_4$.

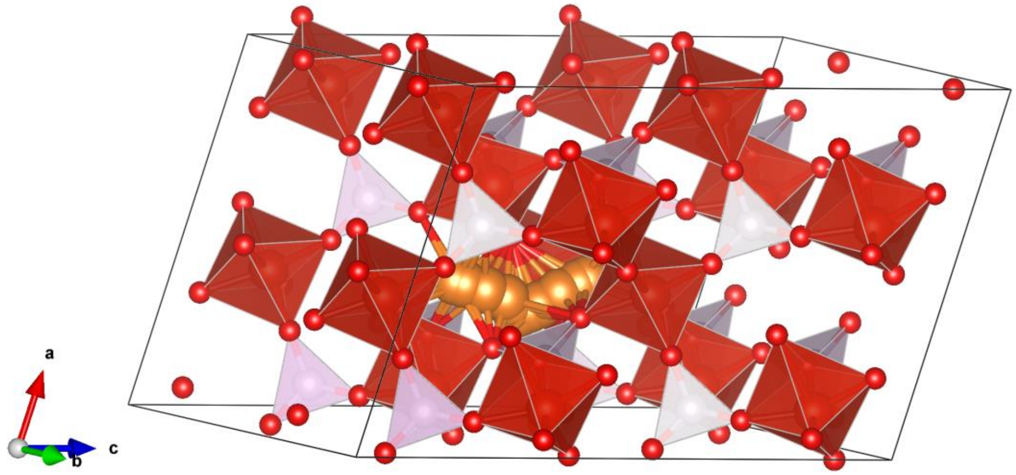

Figure S8. Visualization of the NEB path in ε-$VOPO_4$.

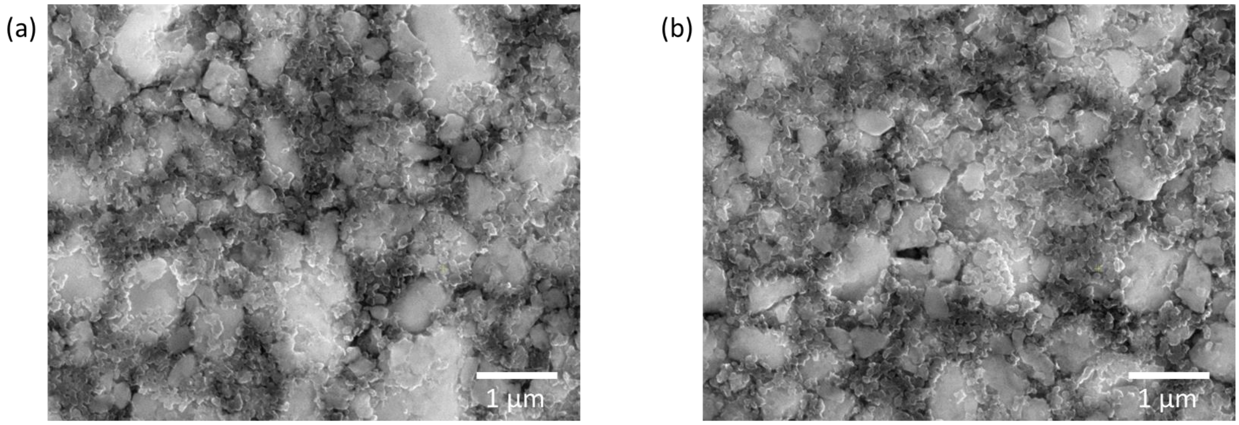

Figure S9. SEM images of the ε-$VOPO_4$ electrodes (a) before and (b) after Li pre-cycling.
